# Use of Microfluidics to Prepare Lipid-Based Nanocarriers

**DOI:** 10.3390/pharmaceutics15041053

**Published:** 2023-03-24

**Authors:** Alicia Vogelaar, Samantha Marcotte, Jiaqi Cheng, Benazir Oluoch, Jennica Zaro

**Affiliations:** Department of Pharmacology and Pharmaceutical Sciences, USC Alfred E. Mann School of Pharmacy and Pharmaceutical Sciences, University of Southern California, Los Angeles, CA 90089, USA

**Keywords:** lipid-based nanoparticles, liposomes, lipid nanoparticles, solid lipid nanoparticles, microfluidics

## Abstract

Lipid-based nanoparticles (LBNPs) are an important tool for the delivery of a diverse set of drug cargoes, including small molecules, oligonucleotides, and proteins and peptides. Despite their development over the past several decades, this technology is still hindered by issues with the manufacturing processes leading to high polydispersity, batch-to-batch and operator-dependent variability, and limits to the production volumes. To overcome these issues, the use of microfluidic techniques in the production of LBNPs has sharply increased over the past two years. Microfluidics overcomes many of the pitfalls seen with conventional production methods, leading to reproducible LBNPs at lower costs and higher yields. In this review, the use of microfluidics in the preparation of various types of LBNPs, including liposomes, lipid nanoparticles, and solid lipid nanoparticles for the delivery of small molecules, oligonucleotides, and peptide/protein drugs is summarized. Various microfluidic parameters, as well as their effects on the physicochemical properties of LBNPs, are also discussed.

## 1. Introduction

Lipid-based nanoparticles (LBNPs), defined as 10–1000 nm nanoparticles composed of lipids as the main structural component, are versatile drug delivery systems (DDSs) as they can encapsulate a variety of drug cargos and selectively target cell/tissues by modification of the outer components. These types of nanosystems, including lipid nanoparticles (LNPs), liposomes, solid lipid nanoparticles (SLNs), and nanostructured lipid carriers (NLCs), have the advantages of protecting the cargo from degradation, allowing lower doses/toxic side effects, enhancing the solubility and therefore bioavailability of hydrophobic molecules, and being biocompatible/biodegradable [1]. Although LNPs were recently brought into the spotlight worldwide due to the emergence of the Pfizer/BioNTech and Moderna COVID-19 mRNA vaccines, lipid-based delivery systems have been studied since the 1960s with the discovery of liposomes. Liposomes are the earliest form of LBNPs and are composed of self-formed closed lipid bilayer vesicles with an aqueous core [2]. This technology has led to the development of many Food and Drug Administration (FDA)-approved drugs, starting with Doxil^®^ in 1995. Due to the complicated production methods, low encapsulation efficiency, and limitations in the physicochemical properties of the drugs that could be incorporated, other types of lipid-based carriers were developed. 

### 1.1. Types of LBNPs and Their Main Components

**Figure 1:**
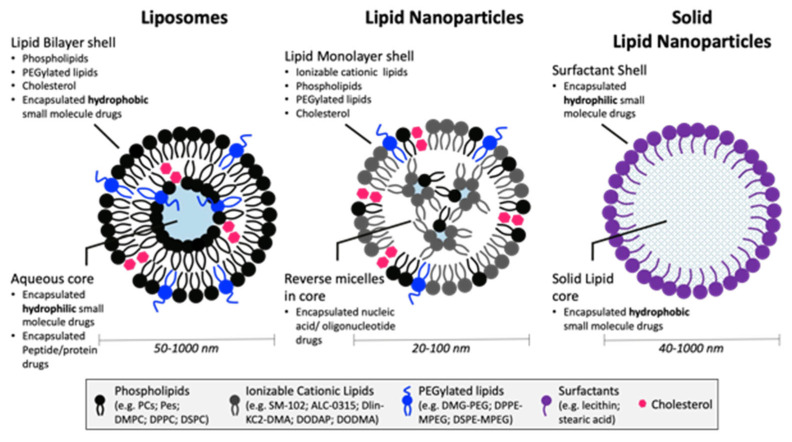
Types of LBNPs utilized in microfluidic production methods.

The main types of LBNPs include liposomes, LNPs, SLNs, and NLCs (Figure 1). Liposomes, the original LBNP system, consist of a unilamellar (single bilayer) or multilamellar shell surrounding an aqueous core. The main components of liposomes are the phospholipids (e.g., phosphatidylcholines, phosphatidylethanolamines, phosphatidylglycerols, and phosphatidylserines) and stabilizers (e.g., cholesterol) which form the lipid bilayer. Liposomes are optimal for the delivery of hydrophilic small molecules, which can be encapsulated in the aqueous core. The stability of liposomes is greatly affected by size, surface charge, and surface modifications, and they also exhibit poor encapsulation efficiency, especially for hydrophobic drugs. SLNs and NLCs, often referred to as second- and third-generation LBNPs, respectively, were developed to overcome many of the issues noted with liposomes. Both SLNs and NLCs contain a surfactant shell, while SLNs have a core matrix of solid lipids, and NLCs have a solid and liquid lipid core. LNPs differ from other LBNPs in their formation of reverse micellar structures contained within the lipid monolayer shell. These differences offer enhanced stability and broaden the ability to encapsulate a variety of drug cargo, including nucleic acids [1,3]. Typically, LNPs are composed of cationic ionizable lipids, polyethylene glycol (PEGylated)-lipids, cholesterol, phosphatidylcholine (PC) lipids, and the drug cargo [4,5]. Cationic ionizable lipids with pK_a_ values below 7 are used to load anionic cargo, such as nucleic acids, at pH values below 7. These cationic lipids are typically composed of tertiary amines linked to 7–18 carbon lipid tails. At normal physiological pH conditions, the complex remains neutral until it is introduced to the lower pH of 5–6 inside the endosome. The protonation of the cationic ionizable lipids in the endosome facilitates disruption of the endosomal membrane and subsequent cargo release into the cytosol [6]. The presence of cholesterol (Chol) increases the stability of the lipid membrane by filling in the gaps between the phospholipids, thereby decreasing membrane fluidity [7]. Additionally, cholesterol greatly enhances nanoparticle stability in the presence of plasma proteins which enhances its circulation half-life [8]. PEGylated-lipids may also be incorporated to increase the stability of LNPs [9,10], control the size and polydispersity [11], prevent aggregation [12], and increase the circulation half-life [13]. PC-lipids also enhance nanoparticle stability [9] and can help enhance cellular delivery [14].

### 1.2. Traditional LBNP Production Approaches

Historically, LBNPs have been produced using a variety of methods, including thin film rehydration techniques, bulk mixing processes, high-pressure homogenization, high-speed stirring, ultra-sonication, microemulsion, solvent emulsification–diffusion, double emulsion, phase inversion temperature, membrane contactor, coacervation, and supercritical fluid-based methods [15]. These processes utilize either high-energy shear stress forces, low-energy chemical or hydrodynamic mechanisms, or nanoprecipitation to form NPs. These techniques rely on the self-formation of LBNPs followed by extrusion through a porous filter, which reduces the yield and increases production costs [16]. Typically, synthesizing LBNPs using traditional methods results in polydispersity with high batch-to-batch variability. Additionally, these manufacturing methods are difficult to scale up past the milliliter volume range, which hinders translation to the clinic. For example, pegylated liposomes that encapsulate Doxorubicin and are clinically available, such as Doxil^®^ or Myocet^®^, require multi-step procedures [17]. In 2011, the FDA suspended Doxil^®^ manufacturing when the company failed to comply with good manufacturing practices (GMPs), likely due to the complicated production process [18]. Effective translation of nanomedicines from the lab to the clinic requires efficient, controllable, and reproducible manufacturing procedures. With a single, continuous step rather than a lengthy, arduous procedure, developing LBNPs via microfluidics can avoid regulatory delays while preserving quality and efficacy.

## 2. Production of LBNPs Using Microfluidics

**Figure 2:**
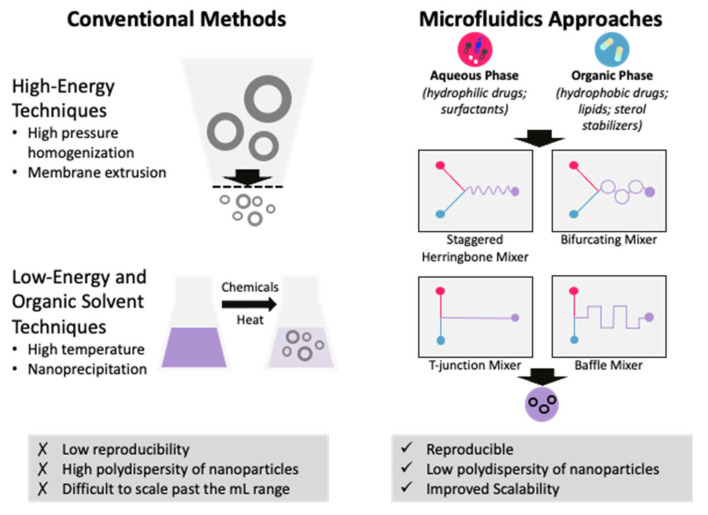
Overview of the microfluidic process to synthesize LBNPs encapsulating drug cargo and comparison with conventional methods.

In response to these issues, microfluidics has quickly become an essential part of LBNP development due to its ease of scaling up, reproducibility, and minimal reagent use [16]. Unlike traditional bulk methods, microfluidics offers an alternative, continuous system to produce LBNPs encapsulating drug cargoes with high reproducibility, size manipulation with simple parameter adjustments, and cost efficiency. The principle of microfluidics involves the manipulation of liquids at micro-scale dimensions using a microfluidic device equipped with a micromixer (Figure 2). Precise control of the mixing of the lipids and drug cargo is managed by injecting solutions of lipids in an organic solvent and the drug cargo in an aqueous solvent into the microfluidic device at specified flow rates. The rapid mixing of the two solutions induced by the micromixer enables the self-formation of LBNPs due to the rapid dilution of the organic solvent. LBNPs produced by microfluidic techniques display reproducible homogenous size distribution and enhanced stability compared to conventional techniques [19]. Importantly, LBNP production can easily be scaled up using the parallelization of devices [20] or using commercially available systems, such as the NanoAssemblr system by Precision Nanosystems [21]. In fact, Pfizer/BioNTech and Moderna Therapeutics both relied on scaled-up microfluidic technology for the mass production of COVID-19 vaccines [22,23,24].

An invaluable benefit of LBNP synthesis by microfluidics is the ability to control particle sizes because the potency of LBNPs is highly dependent on the particle size, but the optimal size varies based on the identity of the target cell and the route of administration. Initial studies by Moderna Therapeutics on the COVID-19 vaccine mRNA-1273 found that maximal potency was achieved with diameters between 75 and 95 nm following intramuscular administration [24]. This is in agreement with stability studies on the Pfizer/BioNTech BNT162b2 COVID-19 vaccine that observed particle sizes around 90 nm [25]. For targeting hepatocytes, generally LNPs ≤100 nm are needed in order to pass through the fenestrae on liver sinusoidal endothelial cells [26]. Interestingly, Chen et al. observed that LNP-siRNA systems targeting Factor VII (FVII) were most potent when the particle diameter was ~45 nm and was administered subcutaneously. However, when administered intravenously, the 78 nm diameter LNP displayed the highest hepatic gene silencing activity [27]. On the other hand, it has been suggested that 200–500 nm sized LNPs are most effective for targeting dendritic cells [11] while much smaller sized LNPs are needed when targeting tumors or the lymphatic system [28]. Consequently, developing LBNP therapeutics involves determining the optimal particle size based on its intended use. Luckily, the minimal reagent use and facile production make the synthesis of LBNPs for screening libraries feasible.

Important microfluidic conditions to consider include the micromixer geometry, total flow rate (TFR), flow rate ratio (FRR), and lipid concentration. Generally, high TFR, FRR, and low lipid concentrations produce smaller-sized LNPs. The effect of micromixer geometry is less defined as it can be case-dependent, but those that induce rapid mixing are preferable for the production of smaller LBNPs.

The most common micromixer geometries used to develop LBNPs for drug delivery include T-junction, baffle, staggered herringbone, and bifurcating mixers (Figure 2). Precision Nanosystems’ instruments are equipped with bifurcating mixers while the commercially available nanoparticle production device, iLiNP, from Lilac Pharma contains a baffle mixer. The iLiNP device has been shown to be able to control LNP size from 20–100 nm in 10 nm increments [29]. Smaller LNPs were homogenously produced using higher TFR and FRR values and lower lipid concentrations. While the same trend was observed for LNPs produced using a staggered herringbone mixer (SHM), the particle size range was 30 to 80 nm at the same conditions. Although both conditions could produce small-sized LNPs with low polydispersity index (PDI) values, larger-size LNPs could not be homogenously produced. Thus, to improve the size distribution of larger LNPs, a three-dimensional iLiNP (3D-iLiNP) device was developed [30]. Similar to the original iLiNP device, the 3D-iLiNP device can control particle size in 10 nm increments by controlling the TFR and FRR. By contrast, the 3D-iLiNP device is capable of producing both small and large LNPs with low PDI values. The advantage of the 3D-iLiNP device was demonstrated by Kimura et al., who compared the luciferase gene silencing activity of 102 nm and 99 nm sized LNPs produced by the 3D-iLiNP and original iLiNP, respectively. Despite their similar sizes, the 3D-iLiNP-prepared LNP suppressed nearly 40% of luciferase expression while the originally produced LNP was limited to 30%. As such, LNP size homogeneity is vital for maximal potency.

## 3. LBNP Drug Delivery Platforms Using Microfluidics

Importantly, LNPs can encapsulate a broad variety of therapeutic agents, including nucleic acids, small molecules, peptides, and proteins. Furthermore, LNPs can encapsulate multiple cargoes to produce a synergistic effect. The aim of this review is to highlight recent studies that have used microfluidics for the production of LBNPs as drug delivery systems (DDSs). The different types of lipids and stabilizers, and their abbreviations, included in this section are summarized in Table 1.

### 3.1. Gene Delivery

Gene therapy, which involves the modulation of gene expression, is a rapidly rising therapeutic platform due to its ability to specifically target disease-causing genes. Due to the Watson–Crick base pairing of RNA or DNA with its target, gene therapies are highly specific and thus alleviate the potential for side effects. However, in order to exert its effect, the nucleic acid must enter the cell, but naked nucleic acids are rapidly degraded in the bloodstream by RNAseS [31] and are unable to enter the cell due to its negative charge [32]. Thus, the use of such DDSs is essential for gene therapy. Out of the different types of LBNPs, LNPs are the most commonly used for gene therapy applications due to the high encapsulation efficiency of nucleic acids. Efforts to optimize LNP systems for nucleic acid delivery led to the development of Onpattro (Patisiran) in 2018, marking the first FDA-approved treatment using small interfering RNA (siRNA), as well as the first FDA-approved LNP system [33]. Onpattro contains a lipid mixture of DLin-MC3-DMA/DSPC/Chol/DMG-mPEG 2000 (50/10/38.5/1.5 mol%), which can be applied for the development of LNPs containing other nucleic acid modalities, including mRNA, pDNA, and siRNA. There are currently many studies that demonstrate the successful application of LNP technology for gene therapy for a broad range of diseases using the Onpattro [34,35,36] or novel lipid formulations. LBNP studies have been greatly facilitated by the application of microfluidic technology. The simple synthesis procedure, minimal reagent use, size control, and reproducibility enabled by microfluidic synthesis of LBNPs allow for the production of mini LBNP libraries for screening. This is particularly helpful when optimizing novel lipid formulations and when determining the ideal nanoparticle size. As mentioned, the FRR is an important determinant of the resulting particle size, and for the purpose of this review, all FRR values are expressed as the aqueous/organic (*v*/*v*) ratio.

#### 3.1.1. siRNA LNPs

One important gene therapy approach is to silence disease-causing genes using small interfering RNA (siRNA). siRNA is a double-stranded 20–24-nucleotide-long RNA and exerts its effect through RNA interference (RNAi), leading to the degradation of its targeted mRNA, and thereby inhibiting protein translation [37]. A summary of recent siRNA-LNP systems developed using microfluidics is shown in Table 2.

In a study by Terada et al., the authors used a design of experiments (DOE) approach to evaluate the effect of preparation parameters (lipid concentration, FRR, and TFR) on the particle size, PDI, and encapsulation efficiency of siRNA-loaded LNPs [38]. The LNPs were produced using an SHM device with lipid components of DODAP/Chol/HSPC/PEG-DSPE (50/10/39/1 mol%) at a concentration of 10–30 mM total lipid. Interestingly, all of the LNPs produced displayed high EEs of around 90% regardless of the parameters used, but the lipid concentration and FRR significantly impacted the particle size and PDI. LNPs produced using lower lipid concentrations and higher FRRs produced smaller LNPs, but it should be noted that FRRs of 3–5 produced similar results. The smallest LNPs produced were with a lipid concentration of 10 mM, FRR of 3, and TFR of 3 mL/min and had a diameter of ~25 nm with a PDI of ~0.15. Overall, the study provides important insights into the design and optimization of LNPs for drug delivery and highlights the value of using a DOE approach for the systematic evaluation of complex formulation variables. However, it is important to note that LNP size is not the only determinant of activity, and the optimal size depends on the target cell. As such, in vivo analysis of LNPs is crucial to optimize the formulation parameters.

In another formula optimization study, Sato et al. investigated the role of a hydrophobic scaffold on the physicochemical properties and activity of siRNA-loaded LNPs [39]. The researchers first compared the firefly luciferase gene silencing activity in HeLa cells stably expressing firefly and Renilla luciferase (HeLa-dluc) of siGL4-loaded LNPs containing the ionizable lipid CL15A6 or CL15H6. All of the LNPs were produced using an SHM microfluidic device at an FRR of 3 and TFR of 1.5 mL/min. Initially, the LNPs were produced with the lipid composition of a pH-sensitive cationic lipid/Chol/DMG-mPEG 2000 at a molar ratio of 50/50/1 or 3. The diameters of LNPs containing 1 mol% and 3 mol% DMG-mPEG 2000 were greater than 50 nm and ~35 nm, respectively. Interestingly, the LNPs prepared with 3 mol% DMG-mPEG 2000 were only active with the inclusion of CL15H6, while both larger LNPs displayed concentration-dependent gene silencing. Correspondingly, the cellular uptake of CL15H6-LNPs was about twice as high as that of CL15A6-LNPs. Next, the authors compared the effects of replacing cholesterol with egg sphingomyelin (ESM). The replacement of cholesterol with ESM reduced the particle size and increased gene silencing activity, producing LNPs with diameters of ~24 nm with an IC_50_ of 7 nM for CL15H6/ESM LNPs compared to 20 nM for CL15H6/Chol LNPs. Since the uptake of ESM- and chol-LNPs was within twofold difference, the authors attribute this increased activity to increased endosomal escape. Next, the authors optimized the molar ratio between CL15H6 and ESM. While LNPs prepared with molar ratios from 40/60 to 70/30 all had similar gene silencing activity (IC_50_ ~10 nM), the diameter was the smallest (22 nm) at a CL15H6/ESM ratio of 40/60. While the size of CL15H6/Chol LNPs could be modified by altering the mol% of DMG-mPEG 2000, the size of CL15H6/ESM-LNPs with reduced or no DMG-mPEG 2000 was unaffected. The authors suggest that this observation is due to ESM stabilizing the LNP surface, thereby negating the need for DMG-mPEG 2000. This study shows that the identity of the ionizable cationic lipid affects LNP potency and replacement of cholesterol with ESM can decrease particle size while increasing potency.

Several studies have shown that smaller-sized LNPs are ideal for targeting the lymph node (LN) [40,41,42]. By targeting the LN, LNPs gain access to major immune cells including dendritic cells (DCs), macrophages, T cells, and B cells [28]. In a study by Nakamura et al., the authors compared the LN transitivity and distribution of 30, 100, and 200 nm sized LNPs (30-LNP, 100-LNP, and 200-LNP, respectively) [28]. LNPs consisting of a mixture of YSK05/Chol/DMG-mPEG 2000 were produced using a microfluidic mixer equipped with an SHM at a TFR of 1.5 mL/min and FRR of 3. The size of the LNPs was controlled by altering the ratio of PEG–DMG, with the 30 nm sized LNP containing the highest mol% DMG-mPEG 2000. Consistent with previous studies, it was observed that only the 30-LNP was capable of efficient LN translocation. To next assess the effect of charge, 30 nm sized LNPs possessing a negative, neutral, or positive charge (Neg-LNP, Neu-LNP, or Pos-LNP, respectively) were synthesized by the addition of DOTAP (a cationic lipid) or CHEMS (an anionic lipid). Interestingly, it was observed that the Neg-LNP had the highest LN penetration. Analysis of LN cells from mice treated with Neg-LNP, Neu-LNP, or Pos-LNP showed that the Neg-LNP had higher cell uptake in B cells and T cells compared to Neu- and Pos-LNP. Overall, this study suggests that small-sized and negatively charged LNPs are ideal for targeting the lymphatic system.

By contrast, larger LNPs (>100 nm) are ideal for targeting dendritic cells (DC) due to increased macropinocytosis activity. To generate siRNA- and mRNA-loaded LNPs intended for DC delivery, Okuda et al. used DOE to assess the effect of TFR, FRR, buffer pH, lipid concentration, the molar ratio of PEGylated-lipid, and salt concentration on particle size [11]. LNPs were produced with a lipid mixture at varying molar ratios of CL4H6, a phospholipid (DOPE, DSPC, or DOPC), Chol, and DMG-mPEG 2000 (4 to 16 mM) using an iLiNP device at a TFR of 0.1 to 0.5 mL/min and FRR of 3 to 9. As expected, decreased TFR, FRR, and %PEG and increased lipid concentrations produced larger LNPs. Notably, increased NaCl concentrations resulted in larger particle sizes and more potent LNPs encapsulating siRNA or mRNA. Of the LNPs generated in this study, the most potent LNPs were produced using CL4H6/DOPE/Chol/DMG-mPEG 2000 (50/10/40/1 mol%) at a TFR of 0.5 mL/min, FRR of 3, lipid concentration of 16 mM, and NaCl concentration of 370 mM. For siRNA-LNPs, LNPs produced using 370 mM NaCl resulted in a 10-fold higher cellular uptake than those produced using 10 mM NaCl, corresponding to ~59% gene silencing activity compared to ~26%. Similarly, the size of mRNA-LNPs produced with 10, 250, or 370 mM NaCl increased with increasing NaCl concentrations, and the largest LNP (195 nm) produced the highest transfection efficiency. This activity was confirmed in vivo in an E.G7-OVA-bearing mouse model using mOVA-loaded LNPs where the largest LNP was capable of shrinking tumor tissues whereas the smaller LNP was limited to reducing tumor growth. It is interesting to note that although increasing NaCl concentrations led to reduced EEs for mRNA-LNPs, the activity of larger LNPs was still more favorable than that of smaller LNPs. Overall, this study highlights the importance of microfluidics for precise size control in order to optimize LNP formulation parameters based on the target cells.

A study by Chen et al. developed LNPs loaded with siRNA against VEGFa in order to enhance cartilage tissue regeneration [43]. LNPs were produced using a microfluidic device with lipid components composed of Dlin-MC3-DMA/Chol/DSPC/DMG-mPEG2000 (50/37.5/10/2.5 mol%) at a TFR of 3. The resulting LNPs had a particle diameter of 71.04 ± 6.18 nm and EE of 87.6 ± 4.5%. In a mouse model of cartilage injury, treatment with VEGFa siRNA LNPs significantly enhanced cartilage formation and reduced angiogenesis in the injured area compared to control groups treated with saline or non-specific siRNA LNPs. This study demonstrates that functional cartilage tissue formation can be promoted by selectively targeting the expression of genes involved in angiogenesis.

**Table 2:** siRNA LNPs for drug delivery and their physicochemical properties.

Composition	TFR (mL/min)	FRR (aq/org)	Mixer	Cargo	EE (%)	Particle Size (nm)	PDI	Zeta Potential (mV)	Administration Route	Reference
DODAP/Chol/HSPC/PEG-DSPE (50/10/39/1 mol%)	3	3	SHM	-	90	~25	~0.15	-	-	[38]
DLin-MC3-DMA/Chol/DSPC/DMG-mPEG2000 (50/37.5/10/2.5 mol%)	-	3	-	siVEGFa	87.6 ± 4.5	71.04 ± 6.18	-	26.34 ± 4.11 mV	SQ	[43]
Proprietary ionizable amino lipid/DSPC/Chol/DMG-mPEG2000 (50/10/38.5/1.5 mol%)	12	3	NanoAssemblr™	siBCR-ABL	>90	55	-	-	IP	[44]
D-Lin-MC3-DMA/DSPC/Chol/DMG-mPEG2000 (50/10/38.5/1.5 mol%)	12	3	NanoAssemblr™ SPARK	siLINC01257	>85	65	0.22	-	-	[34]
DODAP/DSPC/Chol/DSPE-PEG/DiO (50/10/37.5/1.5/1 mol%)	12	3	NanoAssemblr™ SPARK	siSAT1	100	82	0.16	0.18 ± 0.42	-	[45]
CL15H6/ESM/PEG-DMG (39/58/3 mol%)	1.5	3	SHM	siGL4	>90	~22	-	-	-	[39]
YSK05/DOPE/Chol/PEG-SP94/PEG (44/18/26.5/9/2.5 mol%)	0.5	2-4	iLiNP	siMK	>90	60.47 ± 6.9	0.101 ± 0.011	−17.4 ± 5	IV	[46]
DLin-MC3-DMA/DSPC/Chol/DMG-mPEG2000 (50/10/38.5/1.5 mol%)	3	>10	T-junction	siAR-V7/taxane prodrugs	>90	~50	<0.1	~−2	IV	[35]

SHM = staggered herringbone mixer, DODAP = 1,2-dioleoyl-3-dimethylammonium-propane, Chol = cholesterol, HSPC = hydrogenated soy phosphatidylcholine, PEG-DSPE = 1,2-distearoyl-sn-glycero-3-phosphoethanol amine-N-[methoxy-(polyethylene glycol)-2000], DSPC = Distearoylphosphatidylcholine, DMG-mPEG2000 = 1,2-dimyristoyl-rac-glycero-3-methoxypolyethylene glycol-2000, DiO = perchlorate, ESM = egg sphingomyelin, DOPE = 1,2-dioleoyl-*sn*-glycero-3-phosphoethanolamine, SQ = subcutaneous, IP = intraperitoneal, IV = intravenous.

As a potential treatment for chronic myeloid leukemia (CML), Jyotsana et al. targeted the BCR-ABL fusion oncogene [44]. LNPs were produced on the NanoAssemblr™ instrument using a lipid mix containing a proprietary ionizable amino lipid/DSPC/Chol/DMG-mPEG 2000 (50/10/38.5/1.5 mol%). The resulting LNPs had a mean diameter of 55 nm with an EE greater than 90%. The authors observed almost a 100% uptake of the LNPs in K562 cells corresponding to a 90% reduction in BCR-ABL expression levels. LNP treatment of CD34+ primary bone marrow cells from BCR-ABL-positive CML patients showed a higher reduction in BCR-ABL than in healthy donors, suggesting that the LNPs are able to effectively target diseased cells over normal healthy bone marrow cells. Furthermore, using a GFP xenograft leukemia mouse model, the authors observed a 60% knockdown efficiency of BCR-ABL after 10 LNP injections over 35 days. The ability to specifically target an oncogene is an exciting example of just one approach for treating cancer using LNPs.

While typically the siRNA is designed to exert its effect by inhibiting the expression of disease-causing genes to prevent protein translation, long non-coding RNAs (lncRNAs) are emerging as a promising therapeutic target. As a treatment for acute myeloid leukemia (AML), Connerty et al. designed a siRNA-loaded nanoparticle targeting the novel oncogenic lncRNA LINC0125 using the lipid composition of Onpattro (D-Lin-MC3-DMA/DSPC/Chol/DMG-mPEG 2000 (50/10/38.5/1.5 mol%) [34]. LNPs packed with anti-LINC01257 siRNA (LNP-si-LINC01257) were produced with the NanoAssemblr™ instrument using an FRR of 3 and TFR of 12 mL/min. The resulting nanoparticles had a mean diameter of ~65 nm with a PDI less than 0.22 and EE of >85%. The uptake in Kasumi-1 cells was greater than 95%, corresponding to a 55% reduction in total cell count. Importantly, healthy peripheral blood mononuclear cells were unaffected. Importantly, the authors note that the LNPs were produced with high batch-to-batch reproducibility, which is crucial for the development of safe and effective therapies.

Current cancer therapy options are often hindered by subsequent resistance of cancer cells to first-line treatments, but this can be circumvented by targeting the genes associated with resistance. By silencing resistance genes, the cells should be sensitized to chemotherapeutic agents. In an effort to combat the chemotherapy and radiation therapy resistance of glioblastoma (GB) cells, Yathindranath et al. prepared LNPs loaded with siRNA targeting the resistance gene Spermidine/spermine N1-acetyltransferase 1 (SAT1) [45]. LNPs were produced using a lipid composition of DODAP/DSPC/cholesterol/DSPE-PEG/DiO of (50/10/37.5/1.5/1 mol%) with the NanoAssemblr™ benchtop instrument at an FRR of 3 and TFR of 12 mL/min. The resulting LNP-siSAT1 had a hydrodynamic radius of 82 nm, a PDI of 0.16, and an EE of 100%. The authors compared the SAT1 knockdown efficiency of LNP-siSAT1 prepared with nitrogen/phosphate (N/P) ratios of 5, 10, or 15. Although the LNPs prepared at N/P ratios of 5 and 10 did not produce measurable SAT1 KD in human GB cell line U251, the LNP-siSAT1 at a N/P ratio of 15 was capable of ~80% SAT1 KD at 80 nM. To show that SAT1 KD increased sensitivity to chemotherapy and radiation treatment, cells were treated with LNP-siSAT1 followed by treatment with doxorubicin, BCNU, or topotecan, and cell viability was determined using the MTT assay. Although LNP-siSAT1 treatment resulted in only a moderate increased cytotoxic response to chemotherapeutic agents in U251 cells, the sensitization to radiation treatments was much more pronounced. KD of SAT1 followed by radiation exposure resulted in ~15% cell viability as compared to ~45% for the scrambled siRNA control group. Importantly, this sensitization was not observed in human brain microvascular endothelial cells (hCMEC/D3), primary human astrocytes (HA), and murine macrophage cells, ANA-1.

In addition to traditional siRNA-LNP production, the use of microfluidics enables the generation of more complex DDSs that combine LNPs with other techniques to enhance their efficacy. For example, the resistance gene silencing approach has been further explored by the development of LNPs co-encapsulating a chemotherapeutic agent and siRNA. Because the siRNA must be present to increase sensitivity to the chemotherapeutic agent, the codelivery of both compounds to the same cell is vital for effective combination therapy. As a potential treatment for hepatocellular carcinoma (HCC) Younis et al. developed LNPs co-encapsulating the chemotherapeutic agent sorafenib (SOR) and siRNA targeting the midkine (MK) gene, which encodes a cytokine that is upregulated and confers SOR resistance in HCC [46]. The authors used the iLiNP microfluidic device to control the particle size and optimize the YSK05/DOPE/Chol/PEG-SP94/PEG ratio and formulation parameters. Increasing the FRR from 2 to 4 linearly decreased the average particle diameter from 220.14 ± 8 nm to 104 ± 9.5 nm with PDI values of ~0.2, but an FRR greater than 4 increased the PDI to greater than 0.3. After exploring different lipid ratios, the optimized LNPs were composed of YSK05/DOPE/Chol/PEG-SP94/PEG (44/18/26.5/9/2.5 mol%) and had a particle diameter of 60.47 ± 6.9 nm, PDI of 0.101 ± 0.011, SOR EE of 96.5 ± 4.8%, and siRNA EE of 94.5 ± 6.5%. By contrast, LNPs produced using the thin lipid film hydration method had a diameter of 160 ± 9.4 nm, PDI of 0.235 ± 0.035, SOR EE of 91.5 ± 9.8%, and siRNA EE of 77.5 ± 8.5%. Importantly, the microfluidic-produced LNPs showed sustained release of SOR over 60 h, indicating that the nanoparticle constructs were highly stable. In an HCC tumor mouse model, treatment with SOR-siMK LNPs effectively combatted SOR resistance and eradicated HCC in mice by ~85%. Notably, this was achieved using a relatively low SOR dose (2.5 mg/kg), highlighting the ability of LNPs to reduce effective doses and the synergistic effect of combination therapy.

In a similar approach, Chen et al. developed LNPs co-encapsulating SOR and siRNA targeting hypoxia-induced factor (HIF1α) due to its association with SOR resistance. LNPs were produced using an in-house designed microfluidic chip containing three stages with specific channels to control the incubation time of input materials. First, SOR is rapidly mixed with lipid-TAT peptide conjugate (lipTAT) to form a cationic core. In the second stage, siRNA is introduced, and the channels are widened to extend the incubation time in order to facilitate the electrostatic adsorption of siRNA to the cationic core. Finally, the structural lipids (DOTAP/DOPE/Chol/DSPE-PEG) are introduced in the third stage to form LNPs co-encapsulating SOR and siHIF1α (LSS NPs) with EEs of ~100% and ~95%, respectively. Screening of LSS NPs formulated with 20%, 30%, 40%, or 50% DOTAP in Hep3B tumor-bearing mice revealed that 30% DOTAP LNPs had significantly higher in vivo transfection efficacy and tumor-targeted accumulation compared to the three other LNPs, and thus were selected for further analysis. Importantly, the LSS NPs also demonstrated higher antitumor activity than free SOR treatment alone, despite the lower dose of SOR encapsulated. Finally, primary cancer cells from a SOR-insensitive HCC patient were used to construct patient-derived primary cell xenografts in BALB/c nude mice. Compared to the negative control siRNA-loaded LSS NPs (NC-LSS NPs), LSS NPs significantly reduced tumor growth and extended survival time, thereby confirming that silencing HIF1α is vital for increasing sensitivity to SOR. The unique microfluidic chip designed by this group shows the high versatility of microfluidic LNP production and offers a promising tool for the design of future LNPs capable of delivering two or more drug cargoes.

Another example of LNPs co-encapsulating chemotherapeutic agents and siRNA was demonstrated by van der Meel et al. as a treatment for prostate cancer [35]. The authors generated LNPs containing siRNA against the splice variant androgen receptor 7 (AR-V7) and taxane prodrugs using the same lipid mixture as Onpattro in a T-junction device with a TFR >10 mL/min and an FRR of 3. Importantly, the authors demonstrate that the inclusion of the prodrug did not affect LNP physicochemical properties, including particle size, homogeneity, or zeta potential and gene silencing activity; LNPs formulated with 0.1–10% prodrug were all around 50 nm in size, had low PDI values (0.06–0.10), and high siRNA entrapment (>90%). Following systemic administration in prostate cancer xenografted mice, LNPs accumulated in the tumor tissue and significantly reduced AR-V7 levels compared to nontreated controls [35].

#### 3.1.2. mRNA LNPs

In contrast to siRNA, messenger RNA (mRNA) induces protein expression, and has been utilized extensively in LNP design (Table 3). mRNA therapeutics can restore proper cellular function in cases where mutated genes disrupt proper protein translation or encode an antigen to produce neutralizing antibodies, as demonstrated by the Pfizer/BioNTech and Moderna COVID-19 mRNA vaccines. Furthermore, mRNA vaccines can be used for cancer immunotherapy [36] or gene editing using the CRISPR/Cas9 system [47].

In a study comparing the physicochemical properties of LNPs synthesized using the bulk method or an automated microfluidic method, Ayad et al. found that the automated microfluidic synthesis resulted in smaller particle sizes and reduced PDI values [48]. In contrast to typical LNPs, these LNPs consisted of a poly(lactic) acid nanoparticle (PLA-NP) core coated with a DPSC/DOTAP lipid membrane to form a lipoparticle (LP). The LP was then successively coated with mRNA and the cell-penetrating peptide LAH4-L1 (mRNA/LAH4-L1 1/5 or 1/20 *w*/*w*) using a particulate layer-by-layer approach. Previously, the authors synthesized the LP by mixing preformed DSPC/DOTAP liposomes (synthesized using microfluidics) with PLA-NP using an orbital mixer, but this required an intermediate time-consuming step for the purification of the liposome. In an effort to improve the LP synthesis protocol, the authors developed a microfluidic system to synthesize LP_auto_. LP_auto_ was synthesized using the NanoAssemblr benchtop instrument with a DSPC/DOTAP (15/85 mol%) lipid solution at 1.65 mM at a TFR of 12 mL/min and FRR of 2 at 40 °C. Regarding particle size, LP_auto_ displayed smaller values than LP as assessed by DLS (175 nm versus 226 nm) and TEM (126 nm versus 233 nm). TEM also revealed a more homogenous lipid deposition for LP_auto_ than LP, corresponding to a lower PDI value of 0.103 for LP_auto_ compared to 0.124 for LP. This trend was conserved following the adsorption of mRNA and LAH4-L1 for both the 1/5 and 1/20 formulations. The transfection efficiency of LP and LP_auto_ was similar when using a 1/20 mRNA/LAH4-L1 ratio but was significantly lower for LP_auto_ than LP when using a 1/5 mRNA/LAH4-L1 ratio. This is likely due to the lower total concentration of lipids observed for LP_auto_, which reduces the adsorption efficiency of mRNA. It should be noted that the preformed DSPC/DOTAP liposome used for the synthesis of the LP was produced using an initial lipid concentration of 30 mM at 70 °C at an FRR of 3 compared to 1.65 mM at 40 °C at an FRR of 2 for LP_auto_. Thus, the higher transfection efficiency of LP may be attributed to the higher lipid concentration, temperature, and/or FRR used in its formulation. As such, further studies using comparable microfluidic preparation parameters are needed.

While it is recognized that LNP physicochemical characteristics are an important consideration, it is difficult or impossible to predict the bioactivity of LNPs displaying similar properties using in vitro methods. As an in vivo screening method to identify the optimal LNP formulation (F01-F16), Guimaraes et al. used LNPs encapsulating barcoded mRNA (b-mRNA), which is mRNA with a barcoded region in the 3′ untranslated region that can be identified via deep sequencing [49]. A set of 16 LNPs, each encapsulating unique b-mRNA, were synthesized using a lipid solution of C12-200/DOPE/Chol/C14-PEG2000 of varying molar ratios with an SHM microfluidic mixer at an FRR of 3. All of the LNPs had a diameter between 74.42 nm and 90.77 with PDI values of 0.174 to 0.233, and 11 out of the 16 had EEs greater than 85%. Following intravenous administration in mice, the LNP-b-mRNA delivery to different organs could be quantified using deep sequencing. Interestingly, F11-F16, which were formulated using an increasing weight ratio of C12-200:mRNA from 5:1 to 25:1, displayed enhanced delivery with increased weight ratios. Importantly, increased LNP-b-mRNA delivery corresponded to increased LNP-mediated mRNA transfection using mRNA encoding luciferase and erythropoietin (EPO). To assess the effect of the nucleic acid cargo identity on the LNP formulation optimization process, the authors generated LNPs encapsulating barcoded DNA (b-DNA) using the same 16 formulation parameters. A comparison of the 16 b-mRNA LNPs and 16 b-DNA LNPs showed a weak correlation between the delivery of LNPs with identical formulations, highlighting the need to optimize LNP formulation parameters based on the encapsulated cargo. Overall, this study is an excellent proof-of-concept for in vivo screening of LNPs that takes advantage of microfluidics to generate the LNP screening library. Importantly, the high reproducibility enabled by microfluidics allows reliable comparison of LNPs produced using different formulation parameters.

As previously mentioned, larger LNPs (>100 nm) are ideal for targeting dendritic cells due to increased macropinocytosis activity. To generate LNPs intended for dendritic cell delivery, Okuda et al. used DOEs to assess the effect of TFR, FRR, buffer pH, lipid concentration, molar ratio of PEGylated-lipid, and salt concentration on particle size [11]. LNPs were produced with a lipid mixture at varying molar ratios of cationic lipid (CL4H6 or YSK05), a phospholipid (DOPE, DSPC, or DOPC), Chol, and DMG-mPEG 2000 (4 to 16 mM) using an iLiNP device at a TFR of 0.1 to 0.5 mL/min and FRR of 3 to 9. Analysis using definitive screening designs (DSDs) revealed that all six factors significantly contributed to particle size, with NaCl concentration having the largest impact. Increased NaCl concentration, lipid concentration, and buffer pH and decreased TFR, FRR, and %PEG led to the formation of larger LNPs. Increased NaCl concentrations also resulted in more potent LNPs encapsulating siRNA or mRNA. Of the LNPs generated in this study, the most potent LNPs were produced using CL4H6/DOPE/Chol/DMG-mPEG 2000 (50/10/40/1 mol%) at a TFR of 0.5 mL/min, FRR of 3, lipid concentration of 16 mM, and NaCl concentration of 370 mM. For siRNA-LNPs, LNPs produced using 370 mM NaCl resulted in a 10-fold higher cellular uptake than those produced using 10 mM NaCl, corresponding to ~59% gene silencing activity compared to ~26%. Similarly, the size of mRNA-LNPs produced with 10, 250, or 370 mM NaCl increased with increasing NaCl concentrations, and the largest LNP (195 nm, PDI 0.1) produced the highest transfection efficiency. This activity was confirmed in vivo in an E.G7-OVA-bearing mouse model using mOVA-loaded LNPs where the largest LNP was capable of shrinking tumor tissues whereas the smaller LNP was limited to reducing tumor growth. It is interesting to note that although increasing NaCl concentrations led to reduced EEs for mRNA-LNPs, the activity of larger LNPs was still more favorable than that of smaller LNPs. This shows that although the increased presence of NaCl likely disrupted the electrostatic interactions between the cationic lipid and RNA, resulting in a lower EE, the particle size had a greater effect on the activity. Overall, this study highlights the importance of optimizing LNP formulation parameters based on the target cells.

In a subsequent study by the same group, the LNP formulation parameters were further optimized to produce dendritic cell-targeting mRNA-LNPs for use in vaccines [36]. The authors first examined the effect of the molar percentages of cationic lipid (CL), phospholipid (PL), and PEGylated-lipid, type of CL (CL4H6 or CL7H6) or PEGylated-lipid (DMG-mPEG 2000 or PEG-DSG), and NaCl concentration while keeping the TFR at 0.5 mL/min and FRR at 3 on the size and activity of mNluc-loaded LNPs. The resulting 14 LNPs displayed diameters ranging from 88 to 754 nm with a PDI of 0.2 or less except for 2 formulation conditions. Consistent with their previous findings, increased NaCl concentrations had the most significant effect on increasing particle size, and decreased %DOPE and %PEG also significantly increased particle size. These conditions were also optimal regarding LNP-induced Nluc expression, but the identity of the CL had the largest impact on LNP activity, with CL4H6-LNPs inducing higher Nluc expression than CL7H6-LNPs. In splenic DCs, the cellular uptake and Nluc expression increased with the increasing size of LNPs up to 700 nm, after which activity decreased. Based on in vivo screening results of the LNPs, the optimal formulation was determined to be CL4H6/DOPE/PEG-DSG (60/10/1.5) with a NaCl concentration of 400 mM to produce LNPs of 547 nm, a PDI of 0.19, and EE of 89.2%. Notably, this formulation exhibited superior transgene expression compared to two clinically relevant formulations, MC3-LNP (the Onpattro formulation), and RNA-LPX, in an E.G7-OVA tumor-bearing mouse model. Overall, this study shows the practicality of microfluidic preparation of LNPs to reliably produce LNP libraries for screening.

In an effort to produce liver-targeting mRNA-LNPs, Hashiba et al. used a DOE approach to evaluate the effect of formulation parameters on physicochemical properties, gene expression, and liver-specificity [50]. The initial LNP screening library contained 18 formulations with varying identity and mol% of CL (CL4H6 or CL15H6) and PL (DSPC or DOPE), mRNA/lipid ratio, and the mol% of DMG-mPEG 2000. The LNPs were prepared with an 8 mM total lipid solution using an iLiNP device at a TFR of 0.5 mL/min and FRR of 3. It was observed that the identity of the CL, identity of the PL, and %PEG were statistically significant factors for the resulting LNP size, while the CL and PL were the main factors influencing PDI. The CL, PL, and %CL were the three statistically significant main factors influencing Nluc expression in the liver, compared to CL, %CL, and %PEG for Nluc expression in the spleen. The results indicated that LNPs of at least 40 nm are needed for liver targeting, and the inclusion of CL4H6 and DSPC produced large, potent, and homogeneous LNPs while increased %CL maximized gene expression. Additionally, higher PEG/PL ratios lead to higher liver specificity. Interestingly, the mRNA/lipid ratio was not a statistically significant factor for any response. Based on the initial screening results, the next LNP library was produced by varying the %CL, %PL, %DMG-mPEG 2000, and PL identity (DSPC or ESM) while fixing the mRNA/lipid ratio at 18.3 and CL as CL4H6. The main factors influencing particle size were %CL and %PEG, while the %PL and %PEG were the main determinants for Nluc expression in the liver. Consistent with the previous library, higher PEG/PL ratios and lower diameters (to 40 nm) corresponded to increased liver specificity. The resulting optimized LNP (B-13 LNP) was composed of CL4H6/ESM/Chol/DMG-mPEG 2000 at a molar ratio of 60/5/35/1.5. Importantly, the B-13 LNPs localized in the liver and induced therapeutically relevant levels of protein expression following intravenous injection in mice. Overall, this study is another example of the usefulness of microfluidics for generating LNP screening libraries and highlights the importance of optimizing formulation conditions for LNPs based on the target site.

Microfluidic synthesis has also been applied to generate mRNA-loaded LNPs for chimeric antigen receptor (CAR) T cell therapy [51]. Billingsley et al. screened a library of 24 LNPs for CAR mRNA delivery to human T cells to produce CAR T cells. The LNPs each contained a different structural analog of an ionizable lipid/DOPE/cholesterol/C14-PEG (35/16/46.5/2.5 mol%) and were synthesized using an SHM microfluidic device at an FRR of 3. Unfortunately, the TFR, total lipid concentration, and mRNA concentration were not optimized. Initial screening was performed by assessing LNP-mediated luciferase mRNA delivery to Jurkat cells and a leading LNP formulation was identified, referred to as C14-4. Interestingly, LNP size, mRNA dosing concentration, and ionizable lipid pK_a_ did not correlate with enhanced delivery. The C14-4 LNP was then loaded with CAR mRNA in order to assess its transfection efficacy in primary human T cells. As a comparison, electroporated (EP) CAR T cells were also produced as this is the conventional method for engineering CAR T cells. The C14-4 LNPs induced equivalent CAR expression on T cells while substantially reducing the T cell cytotoxicity compared to EP CAR T cells (76% cell viability compared to 31%). In a coplated cancer cell killing assay, C14-4 LNP CAR T cells and EP CAR T cells had comparable cytotoxic activity toward Nalm6 ALL cells. These results highlight the great potential for mRNA-LNPs for T cell engineering that can be optimized using microfluidics to generate LNP libraries.

Prenatal gene therapy is a promising approach for congenital diseases to avoid prenatal or perinatal death or long-term morbidity. To design LNPs for in utero mRNA delivery, Riley et al. screened a library of 14 LNPs containing unique ionizable lipids with varying alkyl tail lengths and polyamine cores [52]. The LNPs were produced using a lipid mixture of ionizable lipid/DOPE/Chol/C14-PEG2000 (35/16/46.5/2.5 mol%) in a microfluidic device equipped with an SHM at an FRR of 3. The top performing LNP, A-3, had a diameter of 116 nm, PDI < 0.3, and EE of 81.3%. Unfortunately, the TFR and total lipid concentration were not reported. As a comparison, two clinically relevant formulations were also prepared, DLin-MC3-DMA and jetPEI, with luciferase mRNA to form MC3.luc and jetPEI.luc. LNP A-3.luc, induced a 45- and 3.5-fold increase in fetal liver luciferase expression compared to jetPEI.luc and MC3.luc, respectively. Additionally, A-3.luc was less toxic than MC3.luc, while the toxicity of jetPEI.luc was unfortunately not explored. Finally, the prenatal delivery of LNP A-3 loaded with human EPO mRNA resulted in dose-dependent EPO expression.

As an alternative to PEGylated-lipids, Nogueira et al. explored the use of polysarcosine (pSar) [53]. To optimize pSar LNPs, the pSar chain length was varied (11, 23, 34, or 65 repeats) and the molar fraction ranged from 0 to 10%. Additionally, three ionizable lipids (DODMA, Dlin-MC3-DMA, and DPL14) were compared. LNPs were produced using a 13.5 mM lipid mixture of ionizable lipid/DSPC/Chol/pSar (40/10/(50 − x)/x) in a Precision Nanosystems microfluidic instrument at an FRR of 3 and TFR of 12 mL/min. As a control, LNPs with DMG-mPEG 2000 in place of pSar were also produced, making a total of 25 LNPs generated for screening. Decreased particle sizes were observed with increased pSar chain lengths and increased pSar molar content. In contrast to LNPs formulated with DMG-mPEG 2000, the activity of pSar LNPs increased with increasing pSar molar content. The most active LNP contained DPL14/DSPC/Chol/pSar_23_ (40/45/10/5 mol%) and had a particle size of 85 nm and PDI of 0.166. When formulated with EPO mRNA, the pSar_23_ LNP induced higher EPO expression with reduced toxicity compared to the most potent DMG-mPEG 2000 LNP.

In an excellent study by Cheng et al., selective organ targeting (SORT) LNPs were designed by supplementing the formulation with permanently cationic, anionic, zwitterionic, or ionizable cationic SORT lipids [54]. Remarkably, LNPs could be tuned to specifically target the liver, lung, or spleen by altering the molar ratio and identity of the SORT lipid. Using the Precision Nanosystems NanoAssemblr at an FRR of 3 and TFR of 12 mL/min, the authors synthesized nearly 100 LNPs composed of an ionizable lipid (5A2-Sc8, DLin-MC3-DMA, or C12–200), phospholipid, cholesterol, DMG-mPEG 2000, and a SORT lipid (DOTAP, 18PA, DDAB, EPC, DOCPe, or DSPC) at varying molar ratios to compare tissue-specific gene editing efficiency. By co-encapsulating Cas9 mRNA and sgRNA, the SORT LNPs were capable of inducing CRISPR/Cas gene editing specifically in the liver, lungs, or spleen. Because CRISPR gene editing can be permanent, minimizing off-target delivery of CRISPR/Cas LNPs is a vital consideration for translation to the clinic. Importantly, this study demonstrates that the addition of a fifth lipid to the typical LNP composition can determine the biodistribution of LNPs based on its charge [54].

LNP systems have also been applied to CRISPR/Cas technology. LNP-mediated co-delivery of Cas9 mRNA and single-guide RNA (sgRNA) was first demonstrated by Miller et al. [47]. As a substitute for the cationic and phosphatidylcholine lipids, they designed a library of 72 zwitterionic amino lipids (ZALs) and identified the leading compound as ZA3-Ep10. The resulting optimized LNP was composed of ZA3-Ep10/Chol/PEGylated-lipid (100/77/1 mol ratio) and prepared in the NanoAssemblr (Precision Nanosystems) with an FRR of 3 and TFR of 12 mL/min. ZA3-Ep10 LNPs encapsulating both Cas9 mRNA and sgRNA against LoxP were capable of inducing tdTomato expression when administered intravenously in mice genetically engineered to contain a Lox-Stop-Lox tdTO cassette.

**Table 3:** mRNA LNPs for drug delivery and their physicochemical properties.

Composition	TFR (mL/min)	FRR (aq/org)	Mixer	Cargo	EE (%)	Particle Size (nm)	PDI	Zeta Potential (mV)	Administration Route	Reference
DSPC/DOTAP (15/85 mol%)	2	12	NanoAssemblr™ Benchtop	eGFP	-	175	<0.15	-	-	[48]
C12-200/DOPE/Chol/C14-PEG2000 (35/16/46.5/2.5 mol%)	3	-	SHM	b-mRNA	87.4	83.36	<0.15	−3.77	IV	[49]
CL4H6/DOPE/Chol/DMG-mPEG200 (50/10/40/1 mol%)	0.5	3	iLiNP	mOVA	76.0 ± 5.9	195	<0.15	0.7 ± 0.4	IV	[11]
CL4H6/DOPE/PEG-DSG (60/10/1.5 mol%)	0.5	3	iLiNP	mNluc	89.2	547	<0.15	−1.4	IV	[35]
CL4H6/ESM/Chol/DMG-mPEG200 (59/5/34.5/1.5 mol%)	0.5	3	iLiNP	mNluc	95.5	63.9	<0.15	8.36	IV	[50]
C14-4/DOPE/Chol/C14-PEG (35/16/46.5/2.5 mol%)	-	3	SHM	mNluc	86.3	65.19 ± 0.83	<0.15	-	-	[51]
Ionizable lipid/DOPE/Chol/C14-PEG2000 (35/16/46.5/2.5 mol%)	-	3	SHM	mNluc	81.3	116	<0.15	-	IUT	[52]
DPL14/DSPC/Chol/pSar23 (40/45/10/5 mol%)	12	3	Precision Nanosystems	mNluc	-	85	<0.15	-	IV	[53]
ZA3-Ep10/Chol/PEG-lipid (56/43/1 mol%)	12	3	Precision Nanosystems	Cas9 mRNA/sgLoxP	-	-	<0.15	-	IV	[47]
246C10/DOPE/Chol/PEG-lipid (26.5:20:52:1.5 mol%)	12	-	NanoAssemblr™ Benchtop	Cas9 mRNA/sgAT	92.2	75.3	0.082	-	IV	[55]

SHM = staggered herringbone mixer, DODAP = 1,2-dioleoyl-3-dimethylammonium-propane, Chol = cholesterol, PEG-DSPE = 1,2-distearoyl-sn-glycero-3-phosphoethanol amine-N-[methoxy-(polyethylene glycol)-2000], DSPC = Distearoylphosphatidylcholine, DMG-mPEG2000 = 1,2-dimyristoyl-rac-glycero-3-methoxypolyethylene glycol-2000, ESM = egg sphingomyelin, DOPE = 1,2-dioleoyl-sn-glycero-3-phosphoethanolamine, DOTAP = Dioleoyl-3-trimethylammonium propane, PEG-DSG = 1,2-distearoyl-rac-glycero, methoxyethyleneglycol 2000 ether, IV = intravenous, IUT = in utero transplantation.

As a treatment for hemophilia, Han et al. developed LNPs encapsulating Cas9 mRNA and sgRNA against antithrombin (AT) [55]. LNPs were formulated using 246C10/DOPE/Chol/PEGylated-lipid (26.5/20/52/1.5 mol%) in the NanoAssemblr Benchtop Instrument at a TFR of 12 mL/min. Unfortunately, the FRR was not specified. To maximize the EE and minimize the PDI, the optimal buffer conditions were found to be 7 mM citrate with 20 mM NaCl, resulting in LNPs with an EE of 92.2%, a particle size of 75.3 nm, and a PDI of 0.082. Since hemophilia A is caused by an inversion of the 22nd intron of the *FVIII* gene (F8I22I) while hemophilia B is caused by the loss of function of the *FIX* gene (F9Mut), mice with F8I22I and F9Mut were used for in vivo analysis. Notably, three consecutive treatments of LNP-CRISPR-mAT reduced AT expression by approximately 40 and 70% in the F8I22I and F9Mut mice, respectively. Importantly, LNP-CRISPR-mAT treatment did not produce any off-target effects. Since the main limitation of other gene delivery systems, such as an adeno-associated virus (AAV), is immunogenicity, mice were also treated with AAV-Cas9 in order to compare the immune responses. While AAV-Cas9 treatment elevated anti-Cas9 IgG levels, LNP-CRISPR-mAT treatment did not produce anti-Cas9 IgG antibodies and did not induce cellular immune responses. As such, CRISPR/Cas9 LNP systems are a promising alternative for utilizing CRISPR/Cas9 as a gene editing tool, especially due to the ease of optimizing LNP formulation parameters by microfluidic technology.

#### 3.1.3. pDNA LBNPs

Additionally, there are many recent papers focused on developing and optimizing lipid/polymer hybrid nanoparticles for plasmid DNA (pDNA) delivery (Table 4). Similar to mRNA, pDNA induces protein expression. Upon entering the nucleus, pDNA is transcribed and translated to produce the encoded protein [56]. Compared to mRNA and siRNA, the larger size of pDNA typically results in larger LBNPs with lower EEs. Interestingly, some studies have found that lower TFRs produce smaller LBNPs, suggesting that pDNA LBNPs may have different optimal microfluidic parameters than those encapsulating mRNA or siRNA.

To optimize the microfluidic synthesis of DNA-loaded LNPs and resulting transfection efficiency, Quagliarini et al. explored the effects of altering formulation parameters, including PEGylation, TFR, concentration, and particle density at the cell surface [59]. Consistent with other studies, they found that PEGylation is crucial for producing small, homogeneous LNPs. Surprisingly, it was also observed that the higher TFR (8 mL/min) led to larger particle sizes (178 nm) and PDI values (0.362), resulting in LNPs with lower transfection efficiency in HEK293 cells. On the other hand, using a TFR of 2 mL/min produced LNPs with a particle size of 145 nm and PDI of 0.113 that corresponded to higher transfection efficiency and lower cytotoxicity than Lipofectamine TM 3000, the industry-recognized gold standard in lipid transfection.

In a subsequent study by the same group, Cui et al. focused on the development of LNPs as a delivery system for DNA vaccines against cancer by encapsulating pVAX-hECTM, a DNA vaccine against the human oncogene HER2 [57]. LNPs were produced using the NanoAssemblr Benchtop instrument at a TFR of 2 mL/min and FRR of 3 using a lipid mixture of DOTAP/Dc-Chol/DOPE/DOPC (25/25/25/25 mol%) and a lipid/DNA weight ratio (Rw) of 5, 10, or 20 to produce LNP_5_, LNP_10_, and LNP_20_, respectively. Analysis of the resulting LNPs showed that an Rw of at least 10 is needed to form small, homogenously distributed LNPs. LNP_10_ and LNP_20_ exhibited particle sizes between 120 and 130 nm with PDI values of 0.12 and 0.27, respectively, and DNA encapsulation greater than 60%. Cell viability assays using Chinese hamster ovarian (CHO) and HEK-293 cells identified LNP_10_ as the ideal candidate. Subsequent FACS analysis of HEK-293 cells treated with LNP_10_ confirmed that transfected cells had a large expression of the HER2 antigen on the cell membrane.

In order to maximize nucleic acid vaccine efficacy, recent efforts have focused on developing LNPs that target lymph nodes (LNs) due to their importance in the immune system. Zukancic et al. developed LN-targeting LNPs carrying pDNA by using Tween 80 or Tween 20 in lieu of more common PEGylated lipids [58]. In LNP formulations, PEGylated lipids are used to control the surface property. Thus, the authors hypothesized that altering the structure of PEG and its lipid tails could alter the LNP selective organ targeting capacity. To further assess the role of the PEG helper lipid, LNPs were produced using 1.5% or 3% PEG-DSPE/Tween 80/Tween 20 for a total of 6 LNPs. LNPs were formulated with a lipid mixture of Dlin-MC3-DMA/DSPC/Chol/PEG-DSPE or Tween 80 or Tween 20 at a molar ratio of 52:8:37–38.5:3.0–1.5 using an SHM microfluidic chip at a TFR of 8 mL/min. Unfortunately, the FRR was not mentioned. Replacement of PEG-DSPE with Tween 80 or Tween 20 increased the resulting particle size from 80–120 nm to 150–200 nm while maintaining a low PDI (<0.2). The increase in size is likely due to the shorter PEG chain length in Tweens than PEG-DSPE, which has 45 ethylene glycol units while Tweens have 20 units. Tween LNPs also had lower EEs compared to PEG-DSPE LNPs, ranging from ~40–65% compared to 80–90%, respectively. Unsurprisingly, Tween LNPs also had a much lower transfection efficiency than PEG-DSPE LNPs for 1.5% formulations. However, formulations with 3% Tween 20 exhibited very high gene expression in the draining lymph nodes while other organs were largely unaffected. This study highlights the importance of PEGylated lipids in LNP formulations and presents an exciting opportunity to further optimize highly tissue-selective LNPs.

In a study by Santhanes et al., lipid/polymer hybrid nanoparticles containing pDNA encoding red fluorescent protein were formulated and assessed for transfection efficiency [60]. While conventional LNPs and lipid/polymer hybrid nanoparticles are both encased by a lipid outer layer, the hybrid nanoparticle contains a polymer core composed of poly-lactic-co-glycolic acid (PLGA). These nanoparticles were formed by combining a stream of PLGA and DC-cholesterol solution with a separate stream containing pDNA and mPEG_2000_-DSPE in a NxGen Cartridge chip equipped with a toroidal micromixer. The TFR was kept constant at 6 mL/min, while the FRR was set to 3 or 5. In contrast to many other LNP studies, it was observed that the lower FRR produced slightly smaller LNPs with lower PDI values. Furthermore, at an FRR of 5, no pDNA loading was observed while LNPs formulated at the lower FRR achieved EEs as high as 65%. Gel electrophoresis demonstrated structurally intact encapsulated pDNA while transfection efficacy measured 20% in human embryonic kidney cells HEK293T.

In a study by Ripoll et al., the authors explored the impact of FRR and TFR on the resulting LNP size, PDI, and EE [61]. LNPs encapsulating the commercially available gWiz-GFP plasmid were produced using a lipid mixture of MC3/DOPC/Chol/PEGylated-lipid (50/10/38.5/1.5 mol%) in a NxGen Cartridge chip equipped with a toroidal micromixer. Initially, the authors compared LNPs produced using TFR values from 0.4 mL/min to 20 mL/min. It was observed that increasing the TFR up to 4 mL/min reduced the resulting LNP particle size and PDI, after which the particle size plateaued to around 100 nm. Similarly, the EE increased with increasing TFR but reached a maximum of about 80%. Altering the FRR also had a similar impact, with FRRs below 2 resulting in large polydisperse LNPs with low EEs. At an FRR of 3 and above, particle sizes stayed around 100 nm with low PDI values and high EEs.

### 3.2. Small-Molecule LBNPs

Chemotherapy remains a challenging cancer intervention because of poor selectivity and pharmacokinetics upon systemic administration [62,63]. The inability to differentiate between tumor populations and healthy cells results in off-target adverse effects and reduced therapeutic efficacy due to insufficient accumulation at tumor sites [63]. To combat the significant limitations associated with chemotherapeutic agents, LBNPs for these small molecules are rapidly being developed (Table 5). Encapsulation of the cytotoxic drug in LBNPs increases specificity and safety by enhancing bioavailability, protecting from degradation, and reducing toxicity through efficient, targeted delivery [63,64,65]. LBNPs also execute a sustained release of their cargo, avoiding an initial burst effect and prolonging the mechanism of action by maintaining therapeutic concentrations in target cells [66]. Beginning with the approval of Doxil^®^ in 1995, several chemotherapeutic-loaded liposomes have become available on the market [67]. Despite their initial success, however, complex manufacturing procedures complicate scale-up from the laboratory and result in high batch-to-batch variation that diminishes potency and increases costs.

Microfluidic technology has become a popular, robust method for synthesizing hydrophilic or hydrophobic chemotherapeutic encapsulating LBNPs. Several recent studies have employed microfluidics to produce LBNPs that improve chemotherapy’s potential as a cancer therapy. For example, to improve Paclitaxel’s (PTX) targeted delivery and anticancer efficacy, Arduino et al. utilized co-flow geometry microfluidics to develop PTX-loaded SLNs conjugated to the tumor homing peptide, iRGD [68]. The organic phase, composed of 10 mg/mL Cetyl Palmitate, 3 mg/mL DSPE-PEG-maleimide, and 0.75 mg/mL PTX, flowed at a constant TFR of 10 mL/min. In the outer chamber, the aqueous phase, consisting of 2% Pluronic F68, maintained a flow rate of 50 mL/min to achieve an FRR of 1:5. Subsequently, following microfluidic preparation, a thiol conjugation functionalized the SLNs with the iRGD peptide. The final iRGD-PTX-loaded SLNs had an average particle diameter of 137.3 nm, a PDI of 0.279, an anionic zeta potential of −11.2, and an encapsulation efficiency of 31.7%. In U87-MG cell viability studies, compared to free PTX, iRGD-PTX-SLN significantly reduced cell viability (84% vs. 67%) [68]. Similarly, several other studies have also reported the improved anticancer activity of chemotherapy drugs by microfluidic-produced LNPs, highlighting a promising field of cancer nanomedicine [65,68,69,70].

As a DDS for cancer applications, the physicochemical characteristics of LBNPs are critical. The particle size of LBNPs and a homogenous formulation dictate pharmacokinetics, drug loading/release, and overall therapeutic efficacy [71]. As with nucleic-acid-loaded LNPs, microfluidic parameters such as TFR, FRR, and lipid compositions can alter physicochemical characteristics, encapsulation efficiency, and drug release. Current nanomedicines offer high success rates as cancer drug delivery systems due to their ability to penetrate physiological barriers (<200 nm), navigate tumor fenestrae, and achieve passive targeted delivery via the enhanced permeation and retention (EPR) effect [68,72,73,74]. However, the pharmaceutical application of nanoparticles is complex, as several studies highlight the variability of LBNP cell uptake [11,26,27,28,75]. When preparing stable PTX-loaded liposomes, Jaradat et al. varied different TFR (1–3 mL/min) and FRR (1:2 to 1:4) parameters as well as investigated multiple lipid formulations (DMPC, DPPC, DSPC, and DOPC). The shorter acyl chain length lipids, DMPC and DPPC, produced the most optimal liposomes at a TFR of 1 mL/min and an FRR of 1:4. Particle sizes were less than 200 nm, had a polydispersity index below 0.2, and anionic zeta potentials around −6 [63]. Similarly, Gkionis et al. also investigated the two phosphatidylcholines, DMPC and DSPC, to form doxorubicin (Dox)-loaded liposomes via the automated Dolomite microfluidic system. They adjusted the lipid composition by adding cholesterol (30–35% mol) and DSPE-PEG2000-PE and varied the TFR (250–750 µL/min) to evaluate its effect on the liposomes’ overall characteristics [70]. A TFR of 500 µL/min and FRR 1:10 produced LNPs with encapsulation efficiencies of about 80% and drug loading greater than 30%. Drug release of Dox varied depending on the lipid bilayer’s DMPC/DSPC mol content and fluidity. DMPC-based liposomes had better sustained release, releasing up to 45% of Dox within 48 h, compared to DSPC liposomes, which released more than 85% within the first 48 h. Consistent with the release profiles, further cytotoxicity studies on a panel of human breast cancer cell lines revealed that DMPC was slightly less potent than DSPC, although both were less cytotoxic than free Dox. As demonstrated in the above studies, parameters such as TFR, FRR, and lipid composition significantly impacted the size, size distribution, and encapsulation efficiency of chemotherapeutic-loaded LNPs fabricated by the microfluidic technique. Other factors, such as adding lipids with high critical packing parameters, drug concentration, lipid concentration, and surfactants, can further alter LNP characteristics [17,74]. However, with the proper formulation and manufacturing parameters, LNPs can be designed for specific tumor microenvironments to improve their cellular uptake and therapeutic efficacy.

Different device geometries also influence chemotherapeutic-drug-loaded LBNPs. A novel swirl mixer designed by Tomeh et al. can withstand higher TFRs (>300 mL/min), introduces a new controllable processing parameter with an adjustable number of mixing elements (1, 2, or 4), creates three types of flow including laminar flow, turbulent flow, and transitional flow, and allows easy disassembly for cleaning/sterilization. Under the same processing parameters, the swirl mixer outperformed a standard T-mixer with identical channel lengths by producing smaller, more monodisperse LNPs. In further optimization studies, the mixer demonstrated a high production rate, reproducibility, and precise control of particle sizes with low PDI [65]. With the novel device, Dox-loaded LNPs achieved 79.7% encapsulation efficiency, cumulative drug release, and higher anticancer activity compared to free Dox on HCT 116 cells [65].

Dox-umbelliprenin co-loaded PEGylated DSPC liposomes were fabricated via the automated Dolomite microfluidic system and were compared against conventionally prepared thin-film hydration liposomes in terms of physicochemical characteristics (drug loading, stability, and bilayer fluidity) and cytotoxicity on various human breast cancer cell lines [62]. The microfluidic-produced liposomes showed no difference in morphology and were comparable in size, zeta potential, stability, and drug loading capacity. However, the concentration of Dox and lipids used with the microfluidic method was significantly lower than that of the thin film method. Overall, the liposomes were more homogenous in size and shape with the microfluidic method and introduced higher toxicity to the breast cancer cells at a lower concentration [62]. Another study manufactured Dox-loaded lipid-based nanocarriers (LNCs) in a continuous step with rapid microfluidic mixing [17]. Although the Dox-LNC showed no significant difference in cellular uptake, it had higher toxicity and nuclear accumulation than Doxil^®^ in C26 cells. Moreover, it showed better tumor regression without the excessive use of lipids and significantly improved the overall survival rate [17]. Microfluidic technology simplifies the manufacturing process of chemotherapeutic-loaded LNPs for clinical development while maintaining equivalent or superior characteristics and efficacy.

**Table 5:** Small-molecule LBNPs for drug delivery and their physicochemical properties.

Composition	TFR (mL/min)	FRR (aq/org)	Mixer	Cargo	EE (%)	Particle Size (nm)	PDI	Zeta Potential (mV)	Administration Route	Reference
DSPC/DMPC/Chol/DSPE-PEG2000 (16/49/32/3 mol%)	0.5	0.1	Dolomite 5-input chip	Dox-HCl	84	143	0.22	−0.3	-	[69]
DSPC/DMPC/Chol/DSPE-PEG2000 (5/58/34/3)	0.5	0.1	Dolomite 5-input chip	Dox-HCl	88	82	0.13	−2.1	-	[69]
DSPC/DMPC/Chol/DSPE-PEG2000 (11/52/34/3 mol%)	0.5	0.1	Dolomite 5-input chip	Dox-HCl	59	104	0.14	−2	-	[69]
DSPC/DMPC/Chol/DSPE-PEG2000 (16/49/3/32 mol%)	0.5	0.1	Dolomite 5-input chip	Dox-HCl	58	84	0.14	−2.6	-	[69]
DSPC/DMPC/Chol/DSPE-PEG2000 (57/11/29/3 mol%)	0.5	0.1	Dolomite 5-input chip	Dox-HCl	70	110	0.16	−1.6	-	[69]
DSPC/Chol/DSPE—PEG2000 (64/33/3)	0.5	0.1	Dolomite 5-input chip	Dox-HCl	89.4	266	0.22	−2	-	[69]
DSPC/Chol/DSPE-PEG2000-PE (65/31/4 mol%)	0.5	0.1	Dolomite 5-input chip	Dox/Unbelliprenin	74.0 ± 5.8/ 47.0 ± 1.2	227 ± 1	0.20 ± 0.01	−2.5 ± 0.3	-	[62]
DOPA/EPG/DOPE/HSPC:Chol/DSPE-PEG2000 (37.44/9.36/0.47/8.93/1.9 mol%)	12	0.2	NanoAssemblr™ Benchtop	Dox-HCl	62.7	97.5	0.134	-	IV	[17]
DPPC/Chol (67/33 mol%)	20	0.3	Swirl	Doxorubicin	79.7 ± 4	<200	<0.2	>−10	-	[65]
DMPC/Chol (67/33 mol%)	1	0.25	FLUIGENT MFCS™-EZ	Paclitaxel	88	>147	>0.124	−10	-	[63]
DPPC/Chol (67/33 mol%)	1	0.25	FLUIGENT MFCS™-EZ	Paclitaxel	91	<168	0.183	−11	-	[63]
Cetyl palmitate/DSPE-PEG2000 (95/5 mol%)	10	0.25	Co-flow	Paclitaxel	31.7	137.3	0.279	−11.2	-	[67]
Cetyl palmitate/DSPE-PEG2000 (99/1 mol%)	10	0.25	-	Paclitaxel	54	121	0.11	−23	-	[73]

SHM = staggered herringbone mixer, DODAP = 1,2-dioleoyl-3-dimethylammonium-propane, Chol = cholesterol, HSPC = hydrogenated soy phosphatidylcholine, PEG-DSPE = 1,2-distearoyl-sn-glycero-3-phosphoethanol amine-N-[methoxy-(polyethylene glycol)-2000], DSPC = Distearoylphosphatidylcholine, DMG-mPEG2000 = 1,2-dimyristoyl-rac-glycero-3-methoxypolyethylene glycol-2000, DiO = perchlorate, ESM = egg sphingomyelin, DOPE = 1,2-dioleoyl-sn-glycero-3-phosphoethanolamine, EPG = egg phosphatidylglycerol, IV = intravenous.

Furthermore, Roces et al. utilized the Precision NanoSystems NanoAssemblr Benchtop microfluidic system with an SHM to develop a continuous manufacturing procedure for stable Dox encapsulating PEGylated liposomes, 80–100 nm in size, with a PDI less than 0.2, and an EE greater than 90% [67]. To further demonstrate the feasibility of translation, they implemented a microfluidic scale-up system and compared the resulting liposomal product to the clinically approved Doxil^®^. The benchtop and scaled-up versions exhibited similar physicochemical characteristics of 85–100 nm size, >90% encapsulation efficiency, and <0.2 PDI with high reproducibility using the same manufacturing parameters from the laboratory [67].

In addition to chemotherapeutics, microfluidics can encapsulate various other small molecules into LBNPs. Small molecules hold significant advantages as therapeutic interventions with their ability to quickly diffuse through cellular membranes and reach their respective target without inducing an immune response. However, small molecules invoke toxicity, are susceptible to degradation, and can have limited solubility in aqueous environments [76,77]. Encapsulation into LBNPs via microfluidic technology combats these associated challenges, such as avoiding initial burst release toxicity by instituting controllable sustained release [76,77,78,79,80]. Many recent studies have optimized microfluidic process parameters and formulations to achieve the best small-molecule-loaded LNP characteristics depending on their pharmaceutical application.

In an effort to optimize the microfluidic synthesis of LNPs encapsulating ionizable hydrophilic drugs, Lee et al. used a DOE approach to assess the effects of the amount of total lipid, the ratio of phospholipid to cholesterol, the ratio of anionic lipid to neutral lipid, amounts of drug-loaded, solvent polarity, TFR, and FRR on particle size, PDI, EE, and drug-to-lipid ratio (D/L) [80]. As a model drug, they used doxycycline to produce LNPs loaded with doxycycline (LN-Doxy). Regarding the microfluidic variables, LBNPs were produced using TFRs from 2 mL/min to 12 mL/min, with FRRs from 1 to 4 using the NanoAssemblr™ Benchtop instrument. It was found that FRR impacts particle sizes while both TFR and FRR showed significant effects on PDI. Thus, the TFR was kept at 12 mL/min for the following optimization study to keep the PDI below 0.3. The effect of the FRR on PDI was shown to be dependent on the lipid composition. When the ratio of anionic lipid to neutral lipid was 1, increasing the FRR corresponded to increased PDI. However, when the ratio was set to 5, increasing FRR led to decreased PDI. Since a ratio of 1 corresponds to lower anionic lipid concentration, slower mixing may provide sufficient time for electrostatic interactions between the anionic lipid and the ionized doxycycline, which prevents the formation of lipid aggregates. Conversely, the higher anionic lipid concentration present in the ratio of 5 requires rapid mixing to achieve a homogenous solution, and thus a higher FRR is beneficial for lower PDI. A similar trend was observed for the ratio of phospholipid to cholesterol, where higher FRRs resulted in higher PDI values for lower ratios while lower FRRs resulted in lower PDI values for higher ratios. The effect of FRR on the D/L ratio was also evaluated, and it was found that higher FRRs corresponded to higher D/L values. The resulting optimized LNP was produced using a TFR of 12 mL/min, FRR of 4, and a lipid composition of egg phosphatidylglycerol (EPG)/HSPC/Chol/DSPE-mPEG2000 (46.8/9.4/41.9/1.9 mol%), and displayed a particle size of 215.6 ± 23.0 nm, a PDI of 0.055 ± 0.03, an EE of 0.7309 ± 0.03, and a D/L ratio of 0.412 ± 0.015. In a mouse peritonitis model, 100% of the mice treated with LN-Doxy at a dose of 4.5 mg/kg after *S. aureus* infection were successfully rescued [80].

As notable in the increase of published papers in the last five years, microfluidic preparation of small-molecule-loaded LNPs has become increasingly popular. As with clinically available LNPs, encapsulation of chemotherapeutics by microfluidics improves the anticancer activity of the free drug by implementing controlled drug release and increasing cytotoxicity. Even more so, microfluidic LNPs encapsulating small molecules exhibit equivalent characteristics. The platform allows high controllability of particle size in a single-step process, which is necessary for industrial scale-up and cancer drug delivery. Although formulas and microfluidic parameters require optimization, microfluidics can augment the successful translation of LNPs to pharmaceutical applications in the cancer field.

### 3.3. Protein and Peptide LBNPs

The function of proteins and peptides is highly dependent on factors such as structure and stability, and ensuring a sustained function requires optimal delivery methods to target sites. However, several delivery methods are limited by various physicochemical factors, including protein/particle sizes that can allow non-specific interactions, target location accessibility, unintended signaling cascades and associated downstream effects, and pharmacokinetic/pharmacodynamic (PK/PD) issues, among others [81,82]. Among the several advanced methods of optimizing protein delivery, including employing extracellular vesicles such as exosomes to cross intra- and extra-cellular barriers, such as the blood–brain barrier (BBB) [83], engineering LBNPs for delivery of proteins and other biologics is promising. Compared to other types of drug cargoes, including small-molecule- and nucleic-acid-based drugs, there are fewer examples of protein-based LBNPs utilizing microfluidic techniques in the literature.

The conventional production techniques for protein–LBNPs include emulsification, desolvation, complex coacervation, and electrospray [84,85], all of which can limit the stability and function of the proteins and protein drugs as they are prone to denaturation, aggregation, agglomeration, and adsorption [86,87,88]. Microfluidics, on the other hand, has shown precise and improved protein drug delivery and optimized function, with increased protein–LBNP encapsulation efficiencies, stability, and sustained drug release at the target site. Here, we discuss microfluidics for formulating protein–LNPs and their impact on health.

Proteins, as fundamental but complex processing machinery in biological systems, especially for disease targeting, remain underexplored, and microfluidics has been used to further elucidate their structural and biophysical function in terms of protein–protein interactions in controlled microenvironments. Such controlled environments can generate monodispersed protein–lipid nanodroplets, nanotubes, nanofibers, and nanodiscs that can act as independent reactors to develop high-throughput, repeatable, and scalable experiments [86,89,90,91,92,93]. In addition to enabling the effective delivery of protein cargo to intra- and extra-cellular targets, microfluidics enables the physiological study of biological barriers such as the cell membrane through which protein–LNPs cross to reach their targets. The cell membrane, for instance, is made of a phospholipid bilayer that undergoes self-organization, a phenomenon that remains elusive, and microfluidics seems to provide an avenue for real-time investigation [94,95]. Manipulation of pre-programmable microfluidic parameters, including flow rate ratios (FRR), total flow ratios (TFR), cargo capacities, type and functionality of the micro-chip, type and concentration of lipids, and type of organic solvent, viscosities, and interfacial surface tension, are among the important factors in obtaining stable lipid nanostructures and maintaining the biological integrity of the protein cargo [93,96]. Weaver et al. assessed phospholipid self-assembly in the formation of liposomal nanoparticles encapsulating trypsin and albumin as model proteins. The nature, size, and stability of the nanoparticle were assessed with various hydrocarbons that make the lipid bilayer, including short-chained hydrocarbons, [1,2-dimyristoyl-*sn*-glycero-3-phosphocholine (DMPC) and 1,2-dipalmitoyl-sn-glycerol-3-phosphocholine (DPPC)], and long-chained hydrocarbons, [1,2-dis-stearoyl -sn-glycerol-3-phosphocholine (DSPC) and 1,2-dioleoyl-*sn*-glycero-3-phosphocholine (DOPC)]. The short-chained hydrocarbons with reduced steric and physical hindrance resulted in smaller nanoparticles with better encapsulation efficiencies; however, they exhibited a burst release in the initial ~12 h followed by a slow release lasting less than 72 h [96,97]. By contrast, liposomal nanoparticles made of long-chained hydrocarbons exhibited sustained/slower release profiles observed up to 5 days post-encapsulation and initial release, due to the structurally rigid bilayer from the formation of extra van der Waal forces and increased membrane packing [96].

While microfluidic parameters such as FRR and TFR are quite influential in determining resultant peptide or protein–LBNP characteristics that dictate stability, with higher but optimal values causing shear stress and resulting in smaller and stabler nanoparticles, FRR and the nature of the organic solvent used in the formulation are particularly important as they dictate encapsulation efficiencies and release profiles [96,98]. For instance, the concentration of an organic solvent such as methanol, in addition to a shift in the liquid–liquid interface, affects cholesterol tilt in the phospholipid bilayer and subsequently affects the release of the protein cargo from liposomal nanoparticles. Further, for the lipid bilayer to disassemble and release the protein cargo under experimental conditions designed for liposomal stability, the concentration of the organic solvent must be reduced, which has been achieved by increasing the microfluidic FRR, i.e., from an optimal 3:1 to 5:1 [96]. Another important aspect of lipid nanoparticle stability is the surface charge, as assessed by zeta (ζ)-potential, where studies have indicated that neutral liposomes produce more stable nanostructures with higher encapsulation efficiencies than charged liposomes. For example, Colletier et al. showed a 40% entrapment efficiency of acetylcholinesterase, a 75 kDa enzymatic protein, in neutral liposomes compared to anionic liposomes at 20%, suggesting a reduced electrostatic interaction between the polar hydrocarbon heads and the charged side chains of the cationic or anionic protein cargo [96,97,99]. While microfluidics is becoming increasingly relevant as several potential protein drugs experience PK and PD issues [100], particularly in vivo, protein–LBNPs remain unapproved by regulatory bodies such as the FDA. These drugs can potentially benefit from microfluidic technology as it allows for the controlled mixture of these complex components. Since protein drug delivery still experiences shortfalls, even after decades of scientific research, microfluidics is now indispensable and could replace current techniques.

## 4. Conclusions and Future Directions

As demonstrated by a plethora of research groups, microfluidics has revolutionized LBNP development as DDSs by enabling the ability to control and optimize nanoparticles with high reproducibility. Microfluidic-produced LBNPs can efficiently encapsulate a variety of cargos, including nucleic acids, small molecules, peptides, and proteins, ultimately improving their delivery, potency, and efficacy as therapeutics. Additionally, the fabrication of monodisperse batches enhances biodistribution and diminishes potential side effects. In a single-step process, the technology overcomes the typical challenges of large-scale production with conventional methods, such as time-consuming, complicated procedures. These attributes and the ease of scaling up greatly facilitate the transition of LBNPs from the bench to the clinic.

Previously, the cost and complexity of manufacturing microfluidic chips hindered microfluidic techniques. The maximum flow rate capacity that microfluidic chips could withstand also led to relatively low throughput of LBNPs, limiting production yields. However, the rapid rise of microfluidics has led to the production of many commercially available systems and chips, such as Precision NanoSystems instruments, the iLiNP, and Dolomite chips. Regardless, optimal micromixer geometry can be case-dependent, requiring arduous optimization studies. Furthermore, the micro-scale channels are prone to clogging if aggregates form or due to improperly cleaned channels. In response, novel microfluidic systems, such as swirl mixers, are continually being developed and improved for better chip design and operation.

Microfluidic technology enables facile regulation of physicochemical properties, such as particle size and PDI, by altering the FRR and TFR. While microfluidic conditions (including TFR, FRR, mixer geometry, and lipid ratio/concentration) should be optimized for each system to some extent, the formulation of Onpattro has provided an excellent base for many applications. As shown in Table 2, Table 3, Table 4 and Table 5, many studies fail to provide all the pertinent details about the microfluidic conditions used. Given the influence of such conditions on the resulting LBNP characteristics, we hope that all future LBNP microfluidic studies will report all the microfluidic parameters used. Similarly, to facilitate further development of LBNPs, it is vital for studies to report the lipid mixture used, which has been frustratingly neglected by multiple studies. Regardless, microfluidics has greatly enabled LBNP research and has quickly become the gold standard for LBNP synthesis. Future microfluidics research can utilize the above studies as guidance for manufacturing LBNP DDSs. The promising efficacy of microfluidic-fabricated LBNP therapeutics demonstrated in vitro and in vivo highlights the strengths of microfluidic technology and provides insight into the future of LBNP drug delivery.

## Figures and Tables

**Table 1:** LBNP components.

Abbreviation	Definition
***Cationic Lipids***	
C12-200	1,1′-((2-(4-(2-((2-(bis(2-hydroxydodecyl)amino)ethyl)(2-hydroxydodecyl)amino)ethyl)piperazin-1-yl)ethyl)azanediyl)bis(dodecan-2-ol) (pK_a_ 7)
CL	Cationic Lipid
DOTAP	1,2-dioleoyl-3-trimethylammonium-propane (pK_a_ 7.8)
***Ionizable Cationic Lipids***	
CL4 lipids	Ionizable cationic lipids, pK_a_ ~6.3
CL7 lipids	Ionizable cationic lipids, pK_a_ ~5.9
CL15 lipids	Ionizable cationic lipids, pK_a_ ~7.3
DLin-MC3-DMA	Dilinoleylmethyl-4-dimethylaminobutyrate (pK_a_ 6.4)
DODAP	1,2-dioleoyl-3-dimethylammonium-propane (pK_a_ < 7)
YSK05	1-methyl-4,4-bis(((9Z,12Z)-octadeca-9,12-dien-1-yl)oxy)piperidine (pK_a_ 6.5)
***Anionic Lipids***	
DOPA	1,2-dioleoyl-*sn*-glycero-3-phosphate
EPG	Egg phosphatidylglycerol
***Neutral Lipids***	
DMPC	Dimyristoylphosphatidylcholine
DOPC	1,2-dioleoyl-*sn*-glycero-3-phosphocholine
DOPE	1,2-dioleoyl-*sn*-glycero-3-phosphoethanolamine
DPPC	1,2-dipalmitoyl-*sn*-glycero-3-phosphocholine
DSPC	1,2-Distearoyl-*sn*-glycero-3-phosphocholine
HSPC	Hydrogenated soybean phoshatidylcholine
PC	Phosphatidylcholine
***Stabilizing Agents and Lipids***	
Chol	Cholesterol
DMG-mPEG 2000	1,2-dimyristoyl-rac-glycero-3-methoxypolyethylene glycol-2000
DSPE-PEG 2000 Amine	1,2-distearoyl-*sn*-glycero-3-phosphoethanolamine-N-[amino(polyethylene glycol)-2000]
ESM	Egg sphingomyelin
PEG	Polyethylene glycol

**Table 4:** pDNA LBNPs for drug delivery and their physicochemical properties.

Composition	TFR (mL/min)	FRR (aq/org)	Mixer	Cargo	EE (%)	Particle Size (nm)	PDI	Zeta Potential (mV)	Administration Route	Reference
DOTAP/Dc-Chol/DOPE/DOPC (25/25/25/25 mol%)	2	3	NanoAssemblr Benchtop	pVAX-hECTM	>60	120	0.12	-	-	[57]
DLin-MC3-DMA/DSPC/Chol/Tween20 (52/8/37/3 mol%)	8	-	SHM	pNL1.1CMV	~50	~160	<0.2	~−10.7	IM	[58]
DLin-MC3/DOPC/Chol/PEG-lipid (50/10/38.5/1.5 mol%)	4	3	NanoAssemblr Benchtop	gWiz-GFP plasmid	~80	~100	0.07	−13	-	[59]

SHM = staggered herringbone mixer, Chol = cholesterol, DSPC = Distearoylphosphatidylcholine, DOPE = 1,2-dioleoyl-sn-glycero-3-phosphoethanolamine, DOTAP = Dioleoyl-3-trimethylammonium propane, DOPC = Dipalmitoylphosphatidylcholine, PEG = polyethylene glycol, IM = intramuscular.

## Data Availability

No new data was created for this manuscript.

## Abbreviations

**Abbreviation**	**Definition**
DC	dendritic cells
DDS	drug delivery system
DiO	lipophilic carbocyanine fluorophore
DLS	dynamic light scattering
DOE	design of experiments
EE	encapsulation efficiency
FDA	Food and Drug Administration
FRR	flow rate ratio
GMP	good manufacturing practices
iLiNP	invasive lipid nanoparticle production device
LBNP	lipid-based nanoparticle
LN	lymph node
LNP	lipid nanoparticle
LP	lipoparticle
HFF	hydrodynamic flow focusing
mRNA	messenger RNA
NLC	nanostructured lipid carriers
PDI	polydispersity index
pDNA	plasmid DNA
PK/PD	pharmacokinetics/pharmacodynamics
PTX	Paclitaxel
SHM	staggered herringbone mixer
siRNA	small interfering RNA
SLN	solid lipid nanoparticle
SOR	sorafenib
TEM	transmission electron microscopy
TFR	total flow rate
